# A comparison of high-fidelity and virtual reality simulation as assessment tools in undergraduate medical education

**DOI:** 10.1186/s41077-025-00374-y

**Published:** 2025-08-23

**Authors:** Alexandra F. Macnamara, Alan Rigby, Thozhukat Sathyapalan, David Hepburn

**Affiliations:** 1 Hull York Medical School, University of York, York, UK; 2 Leeds Teaching Hospitals NHS Trust, Leeds, UK; 3 Hull York Medical School, University of Hull, Hull, UK

**Keywords:** Simulation, Assessment, Virtual reality, Undergraduate medical education

## Abstract

**Background:**

Simulation is widely used across many aspects of health professions education and, in recent years, has begun to be explored as an assessme

nt tool, particularly in relation to examining technical clinical skills. Although previous research has suggested that simulation may be an effective tool for assessing clinical skills, there is a lack of evidence exploring which form of technology may be a more reliable assessment tool. This crossover study aimed to compare two forms of simulation technology—a high-fidelity manikin and virtual reality, as potential tools for assessing acute clinical care assessment skills.

**Methods:**

The participating students completed two different simulation scenarios: one scenario using a high-fidelity manikin and one using a virtual reality system. The two scenarios were then marked using a checklist created for the research and a global assessment score. The results for each simulation technology were compared with one another and compared with the participants’ medical final summative assessment scores.

**Results:**

Sixteen students participated in the research. The assessment checklist scores from the two technologies were comparable, with no statistically significant difference (*p* = 0.918) and a strong positive correlation between the two (correlation coefficient = 0.665, *p* = 0.005). However, neither simulation technology had a statistically significant correlation with the summative final written examination paper (high-fidelity manikin: correlation coefficient = − 0.25, *p* = 0.927; virtual reality: correlation coefficient = 0.363, *p* = 0.167) or final clinical examination scores (high-fidelity manikin: correlation coefficient = − 0.204, *p* = 0.449; virtual reality: correlation coefficient = − 0.201, *p* = 0.455).

**Conclusions:**

The findings from this research suggest that virtual reality simulation is comparable to high-fidelity simulation when comparing student scores across the two forms of simulation. However, neither method demonstrated a strong correlation with final summative examination outcomes, suggesting that a single scenario assessment using either technology may not provide an appropriate alternative to existing final summative examinations. To better understand the role of simulation in assessment, further research is needed to compare these two simulation technologies in more depth and provide additional evidence to support educators in understanding how they can be best used within health professions education.

**Supplementary Information:**

The online version contains supplementary material available at 10.1186/s41077-025-00374-y.

## Background

Simulation in medical education facilitates the teaching and assessment of a wide range of skills, from communication to acute care skills. By providing a realistic clinical environment, simulation enables the practice of these skills in a safe setting without the risks of patient harm [1]. As its use in health professions education expands, simulation is increasingly being explored as an assessment tool [2].

Simulation-based assessment has the advantage of evaluating clinical skills in a controlled environment while maintaining patient safety [2]. This is particularly important when considering competencies relevant to emergency care, including acute assessment skills and the UK Resuscitation Council “ABCDE” approach [3], where it would not be safe or ethically justifiable for students to be assessed on such skills without sufficient training [4, 5].

Although other forms of assessment, such as written papers or the use of simulated patients, can address ethical and patient safety concerns, the argument for simulation-based assessment is further supported by theoretical frameworks [6–8]. One example is Miller’s pyramid, which provides a useful framework for understanding competency-based assessment in medical education [8, 9]. Miller proposed that assessment should progress beyond evaluating theoretical knowledge (“knows”) to higher levels of application (“knows how”) and direct performance (“shows how”) [9]. Simulation has the potential to align with the upper tiers of Miller’s pyramid by allowing learners to demonstrate their clinical abilities in realistic scenarios, meaning that simulation could be a useful assessment tool that moves beyond simple knowledge recall to a more integrated evaluation of clinical competence [10, 11]. However, the ability of a simulation-based assessment to provide a more comprehensive evaluation of skills could be affected by, or even dependent on, the type of simulation used [10, 11].

Various simulation modalities exist within medical education, including part-task trainers, simulated patients, computer-based simulations, and high-fidelity manikins [12–14]. In addition, extended reality, particularly immersive virtual reality, is an emerging form of simulation technology that has the potential to address some of the limitations of well-established forms of simulation [15, 16]. Although it has been suggested that more authentic and realistic simulation experiences, such as advanced, high-fidelity manikins, may be more appropriate to assess the upper tiers of Miller’s pyramid, there is some debate as to whether technologies such as immersive virtual reality can do so in the same way [10, 11, 17]. However, it can be argued that this is largely dependent on the type of outcome being assessed, with a difference between the ability of virtual reality to evaluate practical or cognitive skills [8, 17].

In addition to considering the level of competence assessed, it is also important to recognize that the validity of assessment outcomes may vary depending on the simulation modality [2, 18]. Despite increasing research on simulation-based assessment, evidence remains limited regarding the comparative utility of different simulation technologies, particularly virtual reality technology [2, 19]. The effectiveness of an assessment tool is often evaluated using van der Vleuten’s utility equation, which considers reliability, validity, cost-effectiveness, educational impact, and acceptability [20]. Reliability refers to the reproducibility of assessment results, whereas validity concerns the extent to which the assessment measures the intended competencies [21]. Others have also suggested that factors such as practicality and authenticity should be included when considering an approach to assessment [20, 22].

Previous studies have demonstrated that high-fidelity simulation appears to have appropriate validity and reliability as a way to assess acute care skills [23–25]. In comparison, although virtual reality shows promise as a more novel form of assessment, for example, in establishing measures of clinical competence [26], it has predominantly been utilized as a method of assessing specific practical skills, such as endoscopy [2]. One previous systematic review conducted by Ryall et al. [2] exploring simulation-based assessments concluded that “further research is required to determine which form of simulation-based assessment is best suited in specific health professional learner situations.”

Our prior research compared high-fidelity manikins and virtual reality with a focus on student perceptions and observational learning, in relation to developing emergency assessment skills within an undergraduate medical curriculum [16, 27]. This study extends that work by directly comparing these technologies as assessment tools for acute care skills in undergraduate medical education for final year medical students. In the context of growing demand for simulation-based summative assessment, there remains a research gap in determining the most effective modality for assessing clinical performance [28]. Understanding how these technologies can be optimally utilised will help to address this gap in the existing evidence and inform best practices for simulation-based assessment in medical education [29, 30].

## Methods

### Study design

This study aimed to compare two forms of simulation, a high-fidelity manikin (SimMan 3G, Laerdal ©) and a virtual reality simulator (Oxford Medical Simulation © used with the Oculus Rift Headset, Meta ©), as assessment tools using a crossover study design. A crossover study design was chosen to minimize inter-participant variability by allowing each student to act as their own control, enhancing comparability between the two technologies [31–33]. The within-subject design is beneficial in simulation-based research, where participant performance may vary significantly depending on prior experience and skill level.

The research was conducted at Castle Hill Hospital, Hull University Teaching Hospitals NHS Trust, during October 2021. This hospital is one of the main clinical teaching bases of the Hull York Medical School and has a dedicated simulation suite in which the research took place.

### Participants and recruitment

All fifth-year medical students were invited to participate in the research through verbal announcements and online advertisements displayed on the students’ virtual learning environment. Full details of the research were provided to fifth-year students in the form of a participant information leaflet.

Students were considered eligible for the research if they were in their fifth and final year of study of their MBBS course at the Hull York Medical School and were sufficiently familiar with both forms of simulation technology. Previous research highlighted how a lack of familiarity with a virtual environment can influence the students’ simulation experience [16]. Therefore, eligibility was limited to students who had already been orientated to both simulation technologies through introductory presentations and a video for the virtual reality system and who had previously used both simulation technologies during at least two simulation teaching sessions. This meant that participating students should have undertaken two previous scenarios and observed several peers undertaking scenarios, on each form of technology. The only exclusion criteria for the study were any medical conditions that could pose a risk when using the virtual reality system, as described in the Oculus Rift health and safety warnings [34]. In total, 20 students volunteered for the research project, all of whom were eligible to participate.

## Materials and methods

Each student completed two different simulation scenarios, one on each technology. Students were allocated into two groups, which determined which simulation technology they would use first. Group A started with virtual reality, followed by the high-fidelity manikin, whereas group B completed their first scenario using the high-fidelity manikin and their second scenario using virtual reality. Block randomization for groups of four to six students [35] was used for this aspect of the study due to the logistics of timings throughout the day and to facilitate the flow of participants from one scenario to the next. The randomization was performed using the online randomisation generator “Research Randomizer©” [35].

Five scenario contexts were chosen from the available pre-programmed Oxford Medical Simulation scenarios. The selected virtual reality scenarios were chosen so they both aligned with the Hull York Medical School curriculum and could be replicated with a high-fidelity manikin. The five chosen scenarios were acute asthma, upper gastrointestinal bleed, meningitis, non-ST elevation myocardial infarction, and postoperative opiate overdose. Each scenario was assigned a number, and students were allocated two different scenarios using a random number generator. The number was regenerated if a duplicate scenario was produced. The allocated scenarios allowed students to undertake two different clinical scenarios, one on each type of simulation technology.

For the scenarios being undertaken with the high-fidelity manikin, students would complete this scenario in a dedicated simulation room designed to replicate the clinical environment, with a monitor to display clinical observations and a “nurse” present in the room, played by a clinical teaching fellow with training and experience in delivering simulation sessions, including playing different roles within scenarios, controlling the scenario and facilitating and debriefing scenarios. The voice for the manikin and control of the clinical observations was provided by another experienced teaching fellow in a separate room through a microphone and dedicated computer. Neither clinical teaching fellows were blinded to the study objectives, but were provided with clear guidance to help provide standardisation. The individual assessing and scoring the scenario was able to watch the scenario in a separate room on a monitor.

The scenarios undertaken using virtual reality technology were undertaken in a different room, with one facilitator present. This facilitator was a clinical teaching fellow experienced in delivering simulation teaching, and they ensured that the scenario and headset were correctly set up and remained in the same room to view and score the scenario to allow them to assist should any technical difficulties arise. The clinical teaching fellow was experienced in delivering virtual reality teaching and was experienced in solving common technological issues. No formal scripting was provided for the facilitator, but guidance was provided to the facilitator and participants to ensure that participating students did not access any additional information or guidance within the scenario that would not be accessible in a scenario using a high-fidelity manikin. The scenarios on the virtual reality system are all pre-programmed to respond to actions completed by the participant.

In addition to the guidance provided for the virtual reality scenarios, efforts were made to standardise the simulation scenarios as much as possible by adhering to pre-determined written guidance for each scenario using the high-fidelity manikin, for example, starting measurements for clinical observations, wording for the presenting complaint for the “patient,” potential scenario pathways, and suggested prompts for the nurse. Although this guidance did not always exactly mirror the same wording or measurements used in the virtual reality system, the focus of the scenarios was undertaking a general acute assessment, regardless of the underlying diagnosis, which was reflected in the guidance and assessment criteria. All scenarios used within the research are routinely used in simulation teaching sessions, in addition to other scenarios, which allowed the research team to compare scenarios and adapt relevant guidance for facilitators prior to the research taking place.

All the scenarios were between ten and fifteen minutes in length. After each scenario, the student would have the opportunity to have a 10-min debrief with the facilitator. In the case of the virtual reality scenarios, this was the facilitator present in the room, and in the case of the scenarios using the high-fidelity manikin, this was undertaken by the individual scoring the scenario.

### Outcome measures

Outcome measures for the study included a numerical score based on a checklist, a global rating score of the participant’s performance, and final medical examination scores.

The scenarios were scored with a standardised checklist created specifically for the research. The checklist was based on other checklists in the literature that have been shown to be reliable in assessment [25, 36]; however, changes were made to ensure that the checklist was appropriate for assessment in this context. The amended checklist was piloted on each included virtual reality scenario to ensure that students could sufficiently demonstrate each component using the technology.

The checklists were grouped according to the types of skill groups being assessed (generic clinical skills, ABCDE examination skills, clinical reasoning and management, and non-technical skills) while taking into account the limitations of the simulation technologies. For example, communication skills cannot be directly observed with virtual reality simulation, as no verbal communication is required; however, this can be assessed by selecting relevant options within the scenario. An example of the checklist for myocardial infarction is provided within the supplementary material.

The checklist allowed for an overall total score to be calculated based on the sum of actions completed from the checklist, as well as the opportunity for examiners to record the time that relevant actions were performed in order to evaluate other important aspects of performance, such as delays in treatment or performing actions out of sequence. Each checklist score was converted into a percentage to allow comparisons to be made.

Although it was felt that having a total score for each participant would be a helpful indicator of performance, it was recognized that this may not reflect factors such as significant omissions, so to take this into account, examiners were also asked to provide a global score of the students’ performance based on A to E examination grades (an A grade reflecting a performance that would be expected from a high performing foundation doctor and an E grade reflecting a performance with significant omissions or concerns). This scoring system emulates the grading system used in the host medical school and was used because of familiarity with the facilitators and assessors. The full details of the A–E examination grading can be found in the supplementary material.

Each checklist was completed by a clinical teaching fellow independent of the research team. All virtual reality scenarios were marked by the same staff member; however, due to staff availability, two clinical teaching fellows shared the role of assessing the high-fidelity manikin scenarios. A proportion of the simulation scenarios were also double-marked by a senior and experienced medical consultant physician and medical school examiner (also independent from the research team) to assess interrater reliability and any effects from “hawk” or “dove” examiners [37]. As only a proportion of scenarios were double-marked, the second marks were only used to assess interrater reliability and were not used for any of the other analyses.

Students’ final examination scores for both the final written paper examination and the final clinical examination were also collected as part of the study to compare the students' simulation scores with their performance in summative assessments within the medical school. The medical school Final Written Paper (FWP) is a closed-book assessment that includes a large number of single best answer questions covering the breadth of medical subject areas within the medical school curriculum. The Final Clinical Physical Examination (FCPE) follows the Objective Structured Long Examination Record (OSLER) format of examination [38] and encompasses several stations where students have 15 min for information gathering and focused physical examination, followed by thinking time and finally a discussion with examiners aimed at assessing clinical reasoning. In most cases, the students see real patients within the examination, although occasionally simulated patients (actors) are used, e.g., in a mental health scenario. Each examination score was converted into a percentage.

### Statistical methods

We calculated that a sample size of 16 participants would provide an 80% probability (i.e., power) that the study would be able to detect a minimal between-group difference of 5.6 units (5% significance, two-tailed). This assumes that the within-student standard deviation of the outcome measure is 5.3 units [39].

The statistical analysis was performed using SPSS software, and the results were based on paired measurements. Due to the distribution of the outcome measures, the Wilcoxon matched-pairs signed-rank test was performed. No subgroup analyses were performed. Missing data are reported within the manuscript but are not considered otherwise in our analyses. In addition to the planned statistical analysis using the Wilcoxon matched-pairs signed-rank test as outlined in the research protocol, the researchers decided to also include a correlation analysis, using Spearman’s rank-order correlation. This was decided following discussions around whether an alternative method of analysis could provide additional value and context for the study. It was felt that a correlation analysis would provide a visual representation and vital additional information, particularly in relation to the simulation methodologies compared with the final examination scores. This decision was made prior to undertaking the additional analysis and therefore was not influenced by any potential results. Intraclass correlation coefficients, calculated using a two-way mixed model with an absolute agreement definition [40], were also incorporated into the statistical analysis to measure interrater reliability.

## Results

### Participants

Overall, 20 participants volunteered to participate in the research study, and 16 participants completed the research. Of the four students who did not complete the research, one student had timetabling conflicts, and three others did not attend with no reason given.

Of the 16 participating students, seven were female (43.75%), and nine were male (56.25%). Further demographic data were not collected as part of the study. All participants met the inclusion criteria for the study, and all provided written consent to participate.

### Missing data

One global score rating was missing from the completed assessment forms (1/32 scenarios).

### Scoring and interrater reliability

The mean and median scores for each scenario across both technologies are displayed in Table 1. The mean checklist score ranged from 70.8 to 87.4%. The upper gastrointestinal (GI) bleed scenario had the lowest average score, which was considerably lower than the other scenarios.

**Table 1:** Average scores across the five clinical scenarios

Scenario	Mean checklist score (%)
Asthma	86.2
Meningitis	83.7
NSTEMI	81.7
Post-op opiate overdose	87.4
Upper GI bleed	70.8

When examining the difference in participant scores over time, there did not appear to be a gradual improvement or decline in scores over time for either of the simulation technologies. The scatterplots displaying results over time are available in the supplementary material.

The scores between the first and second scenarios (on either technology) were also compared to ensure that there was no learning effect from either undertaking the scenario or receiving a debriefing, leading to improved scores on the second scenario. Comparing the average scores between the first and second scenarios, using the Wilcoxon signed-rank test, demonstrated no statistically significant difference between the two (*p* = 0.352).

Of the 32 total scenarios undertaken across both technologies, 25 were marked by a second examiner (78.13%).

For the scenarios that were double-marked, intraclass correlation coefficients (ICCs) were calculated overall for both checklist-scored and global scores and calculated for both types of technology. The results are summarized in Table 2.

**Table 2:** Interrater reliability

Simulator	Score type	ICC	Reliability
High-fidelity manikin	Checklist score	0.773	Good
	Global score	0.776	Good
Virtual reality	Checklist score	0.263	Poor
	Global score	0.259	Poor
Combined overall	Checklist score	0.508	Moderate
	Global score	0.642	Moderate

The overall interrater reliability was considered to be moderate for both the checklist and global scores when using estimates suggested by Koo and Li [40]. However, when considering each technology in isolation, the reliability of the scoring scenarios undertaken on the high-fidelity manikin was estimated to be “Good” for both types of scores compared with the “Poor” reliability for scoring virtual reality scenarios.

### High-fidelity simulation compared with virtual reality

The mean checklist score on the high-fidelity manikin was 80.34% (median score 86.45%, range 41.7–100%), compared with a mean score of 84.81% for the virtual reality system (median score 87.75%, range 39.6–95.8%).

The Wilcoxon matched-pairs signed-rank test was used to compare the checklist scores for the two technologies, which revealed no significant difference among the checklist scores (*p* = 0.918).

**Fig. 1:**
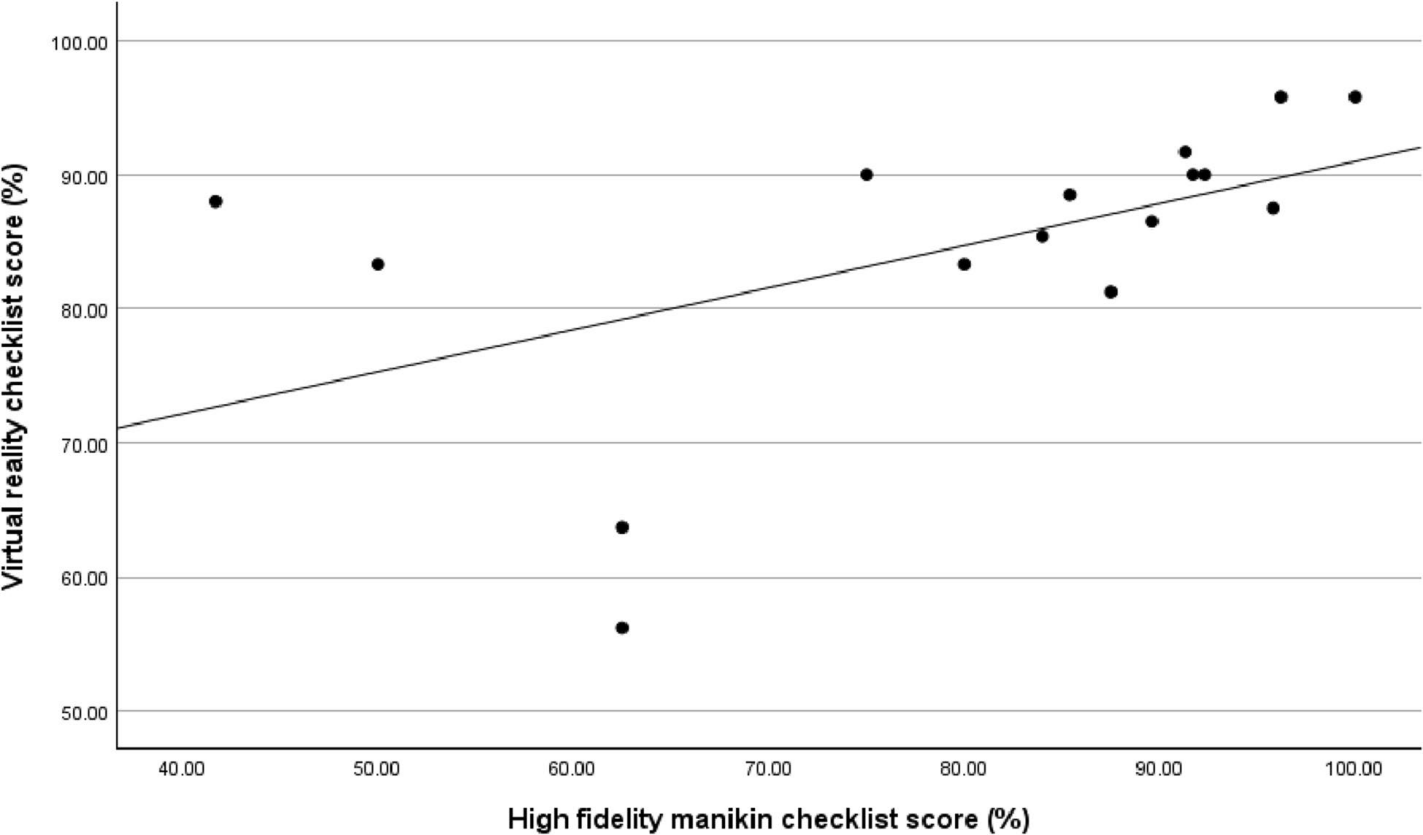
High-fidelity manikin and virtual reality checklist scores

Spearman’s rank-order correlation was performed to analyze the relationship between the students’ checklist scores on the high-fidelity manikin and the virtual reality system. There was a strong positive correlation between the two, which was statistically significant (correlation coefficient = 0.665, *p* = 0.005). There was a notable clustering of scores above 80%, suggesting that students with a higher ranking on a high-fidelity manikin scenario were more likely to also have a higher ranking on a virtual reality scenario (Fig. 1).

To allow for comparisons between the global scores, each score was converted into a numerical value (*A* = 5, *B* = 4, *C* + = 3, *C* − = 2, etc.). The median global score on the high-fidelity manikin was 4.0 (IQR = 2). The median global score on the virtual reality system was 3.0 (IQR = 0.75).

When comparing the median global scores for both technologies using the Wilcoxon signed rank test, the difference was not statistically significant (*p* = 0.66).

### High-fidelity manikin and virtual reality compared with final clinical practice examination scores

The mean final clinical examination practice examination (FCPE) score for the study participants was 72.4% (SD = 7.91).

There was no significant difference between the high-fidelity manikin checklist scores and the FCPE scores (*p* = 0.134). However, there was no statistically significant correlation between the two (correlation coefficient = − 0.204, *p* = 0.449).

When comparing virtual reality scores with FCPE scores, there was a statistically significant difference between the two (*p* = 0.008). According to our correlation analysis, there was no statistically significant correlation between the virtual reality and FCPE scores (correlation coefficient = − 0.201, *p* = 0.455). Figures displaying the correlation between the two technologies and FCPE scores are available in the supplementary material.

### High-fidelity manikin and virtual reality compared with final written paper scores

The mean final written paper (FWP) score was 68.9% (SD = 10.42).

When comparing the checklist scores for the high-fidelity manikin with those from the FWP, there was a statistically significant difference (*p* = 0.034). This was also the case when comparing virtual reality and FWP scores (*p* = 0.001).

The figures displaying correlations between the FWP and the simulation technologies are available in the supplementary material. Neither form of technology had a statistically significant association with the FWP scores (high-fidelity manikin: correlation coefficient = − 0.25, *p* = 0.927; virtual reality: correlation coefficient = 0.363, *p* = 0.167).

## Discussion

Our study aimed to compare two forms of simulation technology (a high-fidelity manikin and virtual reality) in terms of their utility as assessment tools. The two were first compared to determine whether scores from both technologies provided comparable results when the same students were assessed. Both were then individually compared to participants’ final examination scores to analyse whether there was an association between the scores achieved in the simulation scenarios and the final clinical and written assessments.

When considering interrater reliability, a proportion of the scenarios were double-marked by senior examiners within the medical school. The ICC analysis results suggested that interrater reliability was greater when assessing scenarios undertaken on the high-fidelity manikin, with the low interrater reliability for virtual reality scenarios being a significant issue when considering the feasibility of using this technology for summative assessments. There may be multiple reasons for the differences of interrater reliability between the two technologies, including the ability of examiners to see participants physically complete actions using the manikin or a lack of familiarity with virtual reality simulation. Other research exploring virtual reality as a method of assessing clinical competence has shown that interrater reliability varies depending on the observable behaviour [26]. Reliability was not explored in the same depth in this study; however, this could be explored in further detail in future research to help identify any reasons for this and consider how this could be improved to provide greater reliability using virtual reality in assessments.

Our results revealed that the two technologies had broadly comparable results, with a moderate correlation between the two. The visual display of the results also suggests that there was a closer correlation of scores for higher-performing students. However, neither technology had a strong or statistically significant correlation with final examination scores (written or clinical). Although this suggests that neither technology sufficiently allowed candidates to demonstrate their competencies, there may be other factors that could impact the association between the simulation results and the examination results, which are related to some of the limitations of the study.

One important limitation to address is that the scores for each technology were based on the completion of a single scenario, compared with full summative examinations, which would encompass multiple stations, scenarios, or questions. Therefore, aiming to measure competence from a single simulation scenario is somewhat artificial and could affect the validity of the simulation assessments. This finding is further supported by previous research showing that multiple scenarios provide greater reliability [2, 25, 41–43]. This is particularly important when recognising that, when comparing average scores across the five selected scenarios, the upper gastro-intestinal bleed scenario had a notably lower average score than the other four clinical scenarios. This scenario was the least frequently used scenario, with only five students being randomized to this scenario for one of their two scenarios. Although the small numbers could potentially be a contributing factor to the lower score, there is the possibility that the clinical context was more challenging for the participants, which could have impacted the results.

Another study limitation relates to the timing of the simulation scenarios. Although every effort was made to ensure that the scenarios undertaken for the research closely resembled exam conditions, not all the scenarios were capped at exactly 10 min, meaning that some students had slightly longer for their scenarios. This could have had some impact on the scores and introduced another potential source of variation in the scenario scores. In summative exam conditions, more robust ways of ensuring that examiners kept to time would be incorporated to mitigate these possible sources of variation.

The authors also recognise the potential impact of having non-blinded facilitators involved in the simulation scenarios. Because facilitators were aware of the study objectives, their interactions with participants may have introduced unintended bias. Although efforts were made to standardise their roles, this remains a potential limitation.

It should also be noted that the research took place near the start of the academic year, several months before the final examinations, which meant that the students had further opportunities to improve their clinical knowledge and skills prior to undertaking their summative examinations. This could account for some of the differences between the research scenario scores and the final examination scores.

In addition, the risk of volunteer bias may also affect the generalisability of the results. The nature of the research could have appealed more to higher performing students, who may be more likely to take advantage of additional teaching opportunities [44].

Another factor to consider is that the simulation scenarios offer a very different format of examination and arguably test different skills from the existing summative assessments used in the medical school, which do not currently encompass an assessment of acute care skills. The authors initially hypothesised that there may be a correlation between the study simulation scenarios and the final clinical examination in particular, as theoretically more able students may perform well across different forms of assessment. However, when further considering the differences between the two, it is perhaps unsurprising that there was no correlation between the two forms of assessment, particularly given that the results represent only a small, self-selected group of students. Although both the study scenarios and the FCPE scenarios are practical assessments of clinical skills, there are notable differences between the required approach in an acute scenario with emergency management, and history taking and physical examination in patients with long-term conditions that may be well controlled.

A previous systematic review conducted by Ryall et al. [2] concluded that “simulation was more robust when used as an assessment in combination with other assessment tools and when more than one simulation scenario was used.” Although this study has begun to explore two different simulation technologies as assessment tools, in order to further assess the utility of both simulation technologies, additional research should be undertaken that incorporates additional scenarios and other forms of assessment. This would aim to more closely emulate real summative examinations and provide more robust findings of how reliable the two technologies may be. Additional research could also consider the inclusion of medical professionals at different levels of training to assess how the technologies differentiate between those with differing levels of experience. In addition, future simulation-based assessments should aim to incorporate the key principles outlined by Buléon et al. [45], aimed at good practice when implementing simulation-based summative assessments.

## Conclusion

Our research aimed to compare two different forms of simulation technology (a high-fidelity manikin and virtual reality) as assessment tools. Our research findings demonstrated that these two forms of technology were comparable when assessing student simulation scores from both technologies. However, there was no association between the scores from the scenarios on either technology and the final medical school examination scores. This suggests that a single-scenario assessment cannot be considered equivalent to existing final summative examinations. Further research incorporating additional scenarios and published principles of simulation-based assessment may provide additional evidence to help educators understand how different simulation technologies can be best used in assessment for health professions education.

## Supplementary Information

Additional file 1: Table S1. Simulation ABCDE Checklist – 04 Myocardial Infarction (NSTEMI). Table S2. Overview of grading criteria

Additional file 2: Fig. S1. High-fidelity mannikin checklist scores—first to last participant (correlation coefficient = −0.7, *p* = 0.796). Fig. S2. Virtual reality checklist scores—first to last participant (correlation coefficient = −0.204, *p* = 0.449). Fig. S3. Figures displaying the correlation between the two technologies and FCPE scores: High-fidelity manikin and FCPE scores (correlation coefficient = −0.204, *p* = 0.449). Fig. S4. Virtual reality and FCPE scores (correlation coefficient = −0.201, *p* = 0.455). Fig. S5. Figures displaying the correlation between the two technologies and the final written paper (FWP) scores: High fidelity manikin and FWP scores (correlation coefficient = −0.25, *p* = 0.927). Fig. S6. Virtual reality and FWP scores (correlation coefficient = 0.363, *p* = 0.167))

## Data Availability

The datasets used and analysed during the current study are available from the corresponding author on reasonable request.
