# Supplementary material for: A comparison of high-fidelity and virtual reality simulation as assessment tools in undergraduate medical education
Source: Adv Simul (Lond). 2025 Aug 23;10:43. doi: 10.1186/s41077-025-00374-y (PMC12375268; doi:10.1186/s41077-025-00374-y)
Supplement: Supplementary file 1 — Additional file 1: Table S1. Simulation ABCDE Checklist – 04 Myocardial Infarction (NSTEMI). Table S2. Overview of grading criteria [file 41077_2025_374_MOESM1_ESM.zip › Supplementary table 1.docx]

**Supplementary material**

**Supplementary table 1: Simulation ABCDE Checklist – 04 Myocardial Infarction (NSTEMI)**

Student study number: Examiner initials:

| **Task Group** | **Task** | **Time Performed** | **Action completed & timely** | **Not fully complete or untimely** | **Not performed** | **Notes (e.g. technical issues)** |
| --- | --- | --- | --- | --- | --- | --- |
| **Generic Clinical Skill Requirements** | | | | | | |
| Hygiene | Wash Hands/Appropriate PPE |  | 2 | 1 | 0 |  |
| Patient History | Take a brief, focused history |  | 2 | 1 | 0 |  |
|  | Specifically ask about allergies |  | 2 | 1 | 0 |  |
| **ABCDE examination skills** | | | | | | |
| Airway skills | Recognise airway is patent |  | 2 | 1 | 0 |  |
| Breathing skills | Check Respiratory Rate |  | 2 | 1 | 0 |  |
|  | Check Oxygen Saturations |  | 2 | 1 | 0 |  |
|  | Perform full respiratory examination (if technology allows to include: trachea, palpation, percussion and auscultation) |  | 2 | 1 | 0 |  |
| Circulation skills | Check pulse |  | 2 | 1 | 0 |  |
|  | Check blood pressure |  | 2 | 1 | 0 |  |
|  | Check capillary refill |  | 2 | 1 | 0 |  |
|  | Insert intravenous cannula |  | 2 | 1 | 0 |  |
|  | Take bloods |  | 2 | 1 | 0 |  |
| Disability and exposure | Check pupils |  | 2 | 1 | 0 |  |
|  | Check capillary blood glucose |  | 2 | 1 | 0 |  |
|  | Check temperature |  | 2 | 1 | 0 |  |
|  | Full exposure |  | 2 | 1 | 0 |  |
|  | Re-assess at end of ABCDE |  | 2 | 1 | 0 |  |
| **Clinical Reasoning and Management** | | | | | | |
| Specific investigations | Requesting a 12 lead ECG |  | 2 | 1 | 0 |  |
| Specific management | Prescribe GTN |  | 2 | 1 | 0 |  |
|  | Prescribe Aspirin |  | 2 | 1 | 0 |  |
| **Non-technical skills** | | | | | | |
| Communication | Communication with patient/reassurance |  | 2 | 1 | 0 |  |
| Management | Performs tasks in an appropriate order |  | 2 | 1 | 0 |  |
|  | Systematic and avoids fixation errors |  | 2 | 1 | 0 |  |
|  | Clear decision making |  | 2 | 1 | 0 |  |
|  | Re-checks following intervention |  | 2 | 1 | 0 |  |
| **Subtotals** | | |  |  |  |  |
| **Total score** | | | / 50 | |  |  |

**Global Score**

**A B C+ C- D E**
