# Supplementary material for: A comparison of high-fidelity and virtual reality simulation as assessment tools in undergraduate medical education
Source: Adv Simul (Lond). 2025 Aug 23;10:43. doi: 10.1186/s41077-025-00374-y (PMC12375268; doi:10.1186/s41077-025-00374-y)
Supplement: Supplementary file 1 — Additional file 1: Table S1. Simulation ABCDE Checklist – 04 Myocardial Infarction (NSTEMI). Table S2. Overview of grading criteria [file 41077_2025_374_MOESM1_ESM.zip › Supplementary table 2.docx]

**Supplementary material**

**Supplementary table 2: Overview of grading criteria**

| **Adjectival**  **grade** | Descriptor | Criteria |
| --- | --- | --- |
| **A** | Well above expected level | An exceptional performance. All components were performed at a level that would be required to achieve a pass grade. In addition, most components were performed at a level well above the requirements to achieve a pass grade. |
| **B** | Above expected level | A performance that demonstrated all of the components at a level that would be required to achieve a pass grade. In addition, some components were performed at a level of competency that exceeded that expectations required to achieve a pass grade and no components were unsatisfactory. |
| **C+** | At expected level | A performance that demonstrated the components that would be expected to achieve a pass grade. |
| **C-** | Borderline for expected level | A performance that demonstrated most of the components that would be expected to achieve a pass grade, but with a few omissions. None of these omissions would give rise to serious concerns about  performance. |
| **D** | Below expected level | A performance that was below that expected to achieve a pass grade, with many omissions/unsatisfactory elements or a serious omission/ unsatisfactory element that would give rise to concerns about performance. |
| **E** | Well below expected level | A performance that was well below that expected to achieve a pass grade and that would give serious concerns about a student's ability to progress to the next level of the course. |
