# Supplementary material for: A comparison of high-fidelity and virtual reality simulation as assessment tools in undergraduate medical education
Source: Adv Simul (Lond). 2025 Aug 23;10:43. doi: 10.1186/s41077-025-00374-y (PMC12375268; doi:10.1186/s41077-025-00374-y)

**Supplementary figures**

Scatterplots comparing checklist scores over time:

**High-fidelity mannikin checklist scores - first to last participant** (correlation coefficient=-0.7, p = 0.796).

**Virtual reality checklist scores - first to last participant** (correlation coefficient=-0.204, p = 0.449).

Figures displaying the correlation between the two technologies and FCPE scores:

***High-fidelity manikin and FCPE scores*** (correlation coefficient=-0.204, p = 0.449).


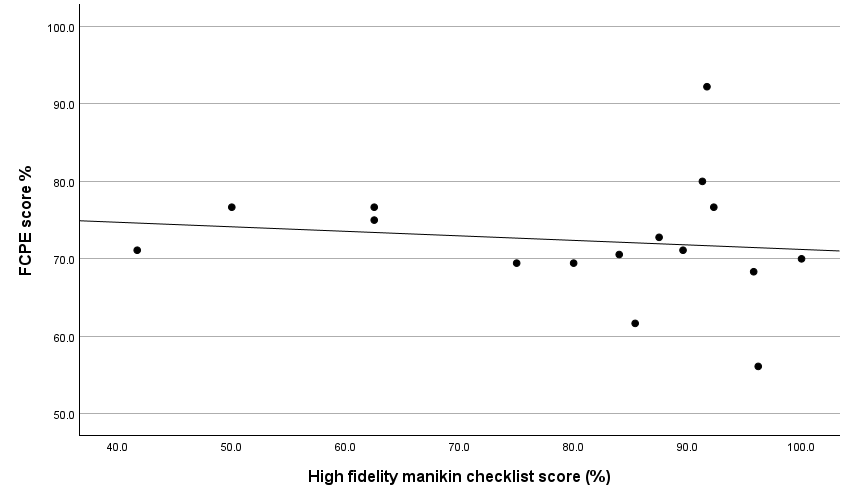


**Virtual reality and FCPE scores** (correlation coefficient = -0.201, p=0.455).
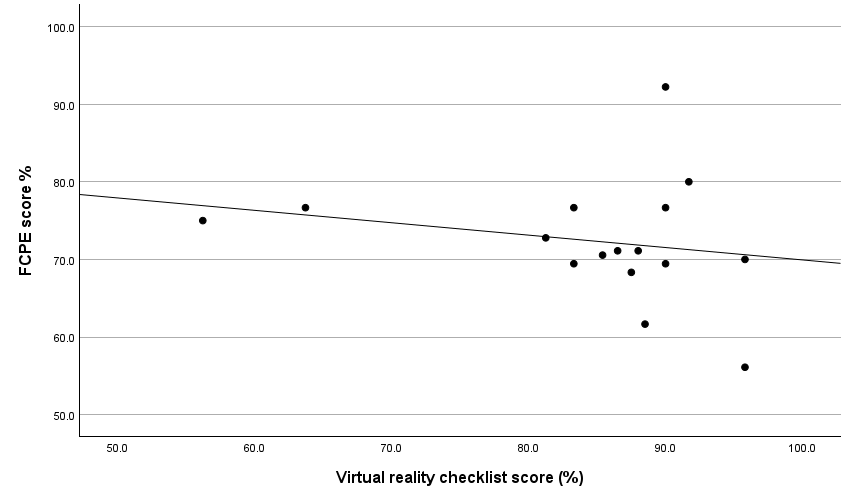


Figures displaying the correlation between the two technologies and the final written paper (FWP) scores:

**
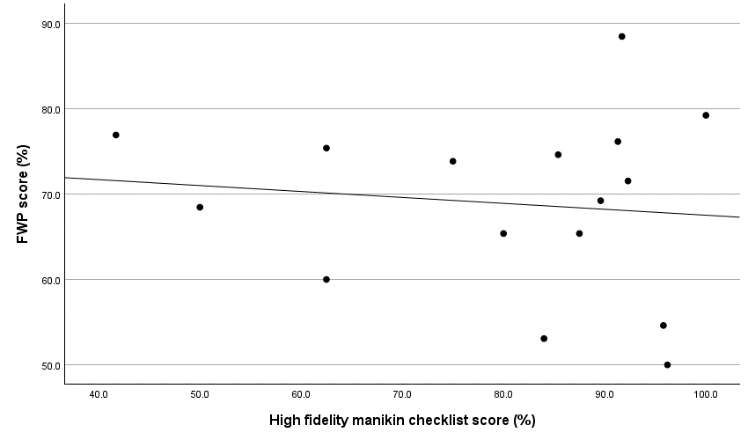
 High fidelity manikin and FWP scores** (correlation coefficient = -0.25, p=0.927)

**Virtual reality and FWP scores** (correlation coefficient = 0.363, p=0.167))


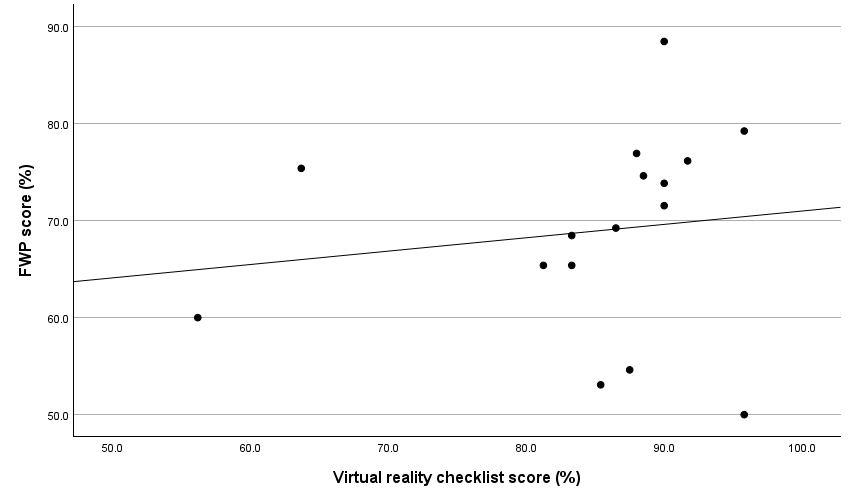

Supplement: Supplementary file 2 — Additional file 2: Fig. S1. High-fidelity mannikin checklist scores—first to last participant (correlation coefficient = −0.7, p = 0.796). Fig. S2. Virtual reality checklist scores—first to last participant (correlation coefficient = −0.204, p = 0.449). Fig. S3. Figures displaying the correlation between the two technologies and FCPE scores: High-fidelity manikin and FCPE scores (correlation coefficient = −0.204, p = 0.449). Fig. S4. Virtual reality and FCPE scores (correlation coefficient = −0.201, p = 0.455). Fig. S5. Figures displaying the correlation between the two technologies and the final written paper (FWP) scores: High fidelity manikin and FWP scores (correlation coefficient = −0.25, p = 0.927). Fig. S6. Virtual reality and FWP scores (correlation coefficient = 0.363, p = 0.167)) [file 41077_2025_374_MOESM2_ESM.docx]
